# Mobile-PolypNet: Lightweight Colon Polyp Segmentation Network for Low-Resource Settings

**DOI:** 10.3390/jimaging8060169

**Published:** 2022-06-14

**Authors:** Ranit Karmakar, Saeid Nooshabadi

**Affiliations:** Electrical and Computer Engineering, Michigan Technological University, Houghton, MI 49931, USA; saeid@mtu.edu

**Keywords:** colorectal cancer, polyp segmentation, deep learning

## Abstract

Colon polyps, small clump of cells on the lining of the colon, can lead to colorectal cancer (CRC), one of the leading types of cancer globally. Hence, early detection of these polyps automatically is crucial in the prevention of CRC. The deep learning models proposed for the detection and segmentation of colorectal polyps are resource-consuming. This paper proposes a lightweight deep learning model for colorectal polyp segmentation that achieved state-of-the-art accuracy while significantly reducing the model size and complexity. The proposed deep learning autoencoder model employs a set of state-of-the-art architectural blocks and optimization objective functions to achieve the desired efficiency. The model is trained and tested on five publicly available colorectal polyp segmentation datasets (CVC-ClinicDB, CVC-ColonDB, EndoScene, Kvasir, and ETIS). We also performed ablation testing on the model to test various aspects of the autoencoder architecture. We performed the model evaluation by using most of the common image-segmentation metrics. The backbone model achieved a DICE score of 0.935 on the Kvasir dataset and 0.945 on the CVC-ClinicDB dataset, improving the accuracy by 4.12% and 5.12%, respectively, over the current state-of-the-art network, while using 88 times fewer parameters, 40 times less storage space, and being computationally 17 times more efficient. Our ablation study showed that the addition of ConvSkip in the autoencoder slightly improves the model’s performance but it was not significant (*p*-value = 0.815).

## 1. Introduction

Colorectal cancer (CRC) is the third leading type of cancer globally, and the second principal cause of cancer-related death in the United States [1]. Approximately 4% of the female and 4.3% of the male population in the United States [2] suffer from colorectal cancer. However, with early detection and proper treatment, 90% of the patients have an increased life span of more than five years [3].

Over the years, different traditional image processing and deep learning networks have been proposed. Although deep learning models outperformed classical image processing [4], they require high computing resources, typically expressed as a frames-per-second (FPS) processing rate (a platform-dependent metric), or the number of floating-point operations (FLOPs) that network executes in order to achieve the task.

This paper develops a deep learning model that produces highly accurate segmentation while being extremely low in resource consumption. This allows the development of image-segmentation tools that could be run on mobile devices in remote locations or in resource limited settings for medical applications.

This paper presents a novel lightweight image-segmentation architecture that is significantly less complex, requiring a fraction of training parameters and a lower number of FLOPs. By using the bottleneck residual blocks on the U-Net [5] backbone, the model was able to achieve a significant reduction in complexity while maintaining high accuracy. The model achieved state-of-the-art performance on the test dataset. The significance of this work is in its novel encoder–decoder architecture backbone that is lightweight and suitable for deployment on mobile devices. We adopted DICE coefficient as objective loss function, which yields more accurate results. We used the same training and testing sets as the current state-of-the-art network, PraNet [4], and performed extensive testing by using important semantic segmentation metrics for better benchmarking.

## 2. Related Work

### 2.1. Traditional Image Processing Techniques

Early works in polyp segmentation proposed the use of handcrafted features. These works mainly focused on two aspects of CRC polyps, shape-based features, and texture or color-based features. The works on shape-based feature detection include edge detection, morphological operations, and shape curvature fitting [6,7,8]. The work on texture-based features includes color wavelet covariance (CWC) [9], discrete wavelet transform (DWT), local binary pattern (LBP) [10], gray level co-occurrence matrix (GLCM) [11], or different combinationsz of these as descriptors [12]. These feature descriptors are then classified by using different classification methods such as linear discriminant analysis (LDA) or support vector machine (SVM).

### 2.2. Cnn Based Methods

In recent years, different deep learning methods have been proposed. Based on the output labels, these networks can be classified into detection- and localization-type networks, and semantic segmentation-type networks.

#### 2.2.1. Localization of Colonal Polyp

The work in [13] proposed a network that first extracts three different types of handcrafted features viz. color, shape and temporal. It next used three different convolutional networks to process features to make binary decisions based on the summation of the output of these networks. Other works [14,15] on detection and localization have explored widely used architectures such as fully convolutional network (FCN) [16], and you-only-look-once (YOLO).

#### 2.2.2. Semantic Segmentation of Colon Polyp

Semantic segmentation has emerged as a preferred technique over localization as it provides more precise information about the polyp, such as its size and shape. Access to multiple publicly available datasets [17,18,19] have facilitated the related investigations. The work in [5] proposed an effective deep learning architecture for biomedical image segmentation that utilizes data augmentation to produce semantic segments. Later works such as residual UNet [20], Unet++ [21] and other networks [22,23,24] have been proposed for semantic segmentation tasks and tested for polyp segmentation. By proposing deeper networks, these works were able to achieve higher accuracy. However, the high accuracy came at a cost—large model size and computational complexity. SFANet [25] introduced a cascade network that utilizes a single encoder block and subsequently uses separate decoder blocks for boundary and area segmentation. Finally, it uses a lighter UNet for the final output. PraNet [4], took a different approach from the encoder–decoder structure and introduced a novel architecture that first predicts the coarse region and then models the boundaries for the final segmentation. The model’s performance has been tested on five different datasets and achieved good performance with high generalizability. However, the model complexity is high, especially for deployment for resource-limited mobile devices.

## 3. Methods

### 3.1. Network Architecture

**Figure 1:**
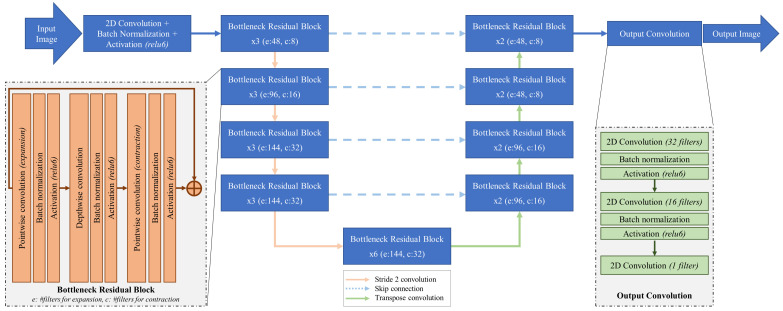
Mobile-PolypNet model backbone architecture with the bottleneck residual blocks and skip connection where x, e, and c in each residual block represent the number of bottleneck residual blocks in each resolution level, number of filters for expansion phase, and number of filters for the contraction phase, respectively.

Our autoencoder model, Mobile-PolypNet (https://github.com/rkarmaka/Mobile-PolypNet (accessed on 13 December 2021)) (Figure 1), uses a similar design philosophy as the original UNet. However, Mobile-PolypNet is significantly different from UNet in its building blocks. The original UNet employs the traditional convolution layer as a building block. Mobile-PolypNet, instead, uses bottleneck residual blocks with depthwise and pointwise separable filters [26]. The building blocks in Mobile-PolypNet have been architectured for the single purpose of significant reduction in computational complexity and memory footprint, while maintaining a similar level of accuracy reported by the state-of-the-art networks.

#### 3.1.1. Input Layer

In Mobile-PolypNet, the input image is first processed by using a traditional convolution layer with 32 filters followed by a depthwise convolution and a pointwise convolution. Batch normalization and Relu6 activation is used after each convolution layer except for the last pointwise convolution layer where a linear activation is used. All the depthwise convolution layers used 3×3 convolution.

#### 3.1.2. Encoder

On each image resolution level Mobile-PolypNet uses three bottleneck inverted residual blocks [26] (see the insert box on the left side of Figure 1). The inverted residual blocks, contrary to the commonly used residual block, first expand the compressed feature map representation to a higher dimension, filter it with efficient depthwise convolution [27], and then project it back to a low-dimensional feature map representation. Stride-2 convolution is used on the first bottleneck residual block to reduce the image dimensions (height and width) by half. The number of expansion filters used in five resolution levels are 48, 96, 144, 144, and 144. We used a contraction factor of 6 for the first 2 levels and 4.5 for the last 3 levels. Also note that each inverted residual block has its own skip connection.

#### 3.1.3. Decoder

Similar to the encoder, the decoder in Mobile-PolypNet uses the bottleneck residual blocks. We use traditional transpose convolution to double the image resolution. Each resolution level contains two bottleneck residual blocks with 96 layers for the bottom two levels and 48 for the top two levels. A contraction factor of 6 was used throughout the decoding path.

#### 3.1.4. Output Layer

The final output from the decoder has eight channels. Rather than directly reducing it down to one channel, we processed the image further by using two traditional convolution layers. First we expanded the image by using 32 filters and then reduced it to 16, and finally to one channel. Each convolution operation was followed by a batch normalization and activation (ReLu6) except for the output layer which uses sigmoid activation without batch normalization.

### 3.2. Network Training

#### 3.2.1. Loss Function

Binary cross-entropy loss, used in UNet, is a standard loss function for semantic segmentation. Although it works well in certain applications, blob segmentation tasks such as polyp segmentation do not give enough global information about the segmented area, making the training difficult. Instead, we have used a negative “DICE” score to evaluate the training loss. The DICE loss is defined as

(1) $$L_{DICE}=\frac{2\sum_{i}^{N}p_{i}g_{i}}{\sum_{i}^{N}p_{i}^{2}+\sum_{i}^{N}g_{i}^{2}}$$

where *p* is the predicted label and *g* is the ground truth label.

#### 3.2.2. Training Setup

All models discussed in this paper have been implemented in TensorFlow with support for graphical processing units (GPU). We have used a platform with NVIDIA GTX 1060 6GB GPU. Input and output both have a size of 224×224. We also used Adam optimizer [28] with a learning rate of $10^{-3}$. The batch size was limited by the available hardware resources and was set to 8, amounting to 979 batches per epoch. After each iteration, the best model was stored, and the training was stopped when validation by DICE score did not improve after 25 epochs.

### 3.3. Statistical Analysis

To compare the similarity and difference between the two results, we performed a two-tailed *t*-test. Statistical analysis of the results was performed in Python by using the SciPy library. We used α=0.05 as our cut-off value for significance testing.

## 4. Experiments

### 4.1. Dataset and Image Preparation

We used the same datasets as the current state-of-the-art model PraNet that reported a significant increase in performance compared to the other available models. The choice of datasets allowed us to do better benchmarking. The training dataset contains 1450 images, with 900 images from the Kvasir dataset and 550 from the CVC-ClinicDB. For the training, we applied data augmentation to achieve a five-fold increase in the size of the dataset; four random rotations between $-90^{\circ }$ and $90^{\circ }$, and one Gaussian blurring. Test images, however, were only resized. Our final training set had 8700 images. For testing, we used hold-out test sets from Kvasir and CVC-ClinicDB, considered as seen, along with CVC-300, CVC-ColonDB, and ETIS, considered as unseen. All the images in the training set and the test set were resized to 224×224 for uniformity.

### 4.2. Settings for the Training and Performance Metrics

For training and validation, we divided the datasets into 90% for training and 10% for validation. We used the validation set to monitor for overfitting. For the model’s performance metrics, we have used the DICE coefficient, mean intersection over union (mIoU), mean absolute error (MAE), and F${}_{\beta }$. We have avoided using frames per second (FPS) as the performance measure as it is a platform-dependent measure. Instead, we used the platform-independent measure, the number of floating-point operations (FLOPs) per image prediction, to measure the model’s computational efficiency.

## 5. Results

**Figure 2:**
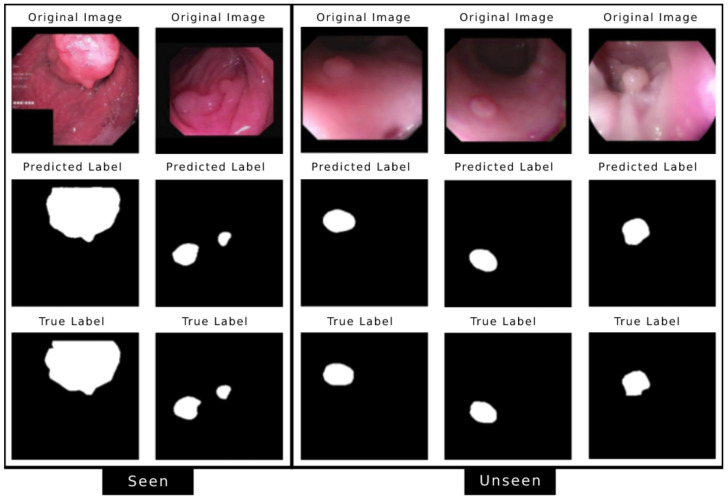
Model’s performance on test images from different datasets (from left) Kvasir, CVC-ClinicDB, CVC-300, Colon-DB, and ETIS where first two are the seen datasets and last three are the unseen datasets.

This section presents our results and the model’s performance on different datasets, seen and unseen. The seen datasets are Kvasir and CVC-ClinicDB, as the model was trained by using the sample images from these datasets. In contrast, the unseen datasets are CVC-300, CVC-ClinicDB, and ETIS, containing images the model has never seen. Figure 2 shows the model’s performance on sample test images from all five datasets.

### 5.1. Accuracy on Individual Dataset

As the model was trained by using sample images from Kvasir and CVC-ClinicDB, we can observe that the model accuracy is very high (Table 1). Except for MAE on the Kvasir dataset, our model outperformed the current state of the art in all evaluation metrics.

### 5.2. Model Generalization

Model generalization is measured by the accuracy of the model on unseen datasets (CVC-300, Colon-DB, and ETIS). Similar to the accuracy on the seen dataset, our model outperformed the state-of-the-art PraNet [4] (Table 2). Similar to PraNet, our model achieved better performance on CVC-300 and Colon-DB compared to ETIS. Images on the ETIS dataset are very different which causes less accuracy.

### 5.3. Model’s Computational Efficiency

In the development of Mobile-PolypNet, major consideration was given to the model’s size and computational efficiency. Table 3 summarizes the number of parameters, disk space required, and FLOPs count, along with accuracy metrics while testing on the Kvasir dataset. The FLOPs counts for the other models have been measured by using TensorFlow with the code provided by the authors. Where the TecsorFlow code was unavailable, we tried to imitate the model by using the information provided by the authors. While outperforming the current state of the art on the accuracy metrics, the proposed model is approximately 83 times smaller in size and about 17 times less computationally expensive compared to PraNet (Table 3).

The PraNet model uses traditional convolution layers with a high number of filters (512, 1024, 2048), resulting in a large number of trainable parameters and FLOPS count. In comparison, Mobile-PolypNet uses separable convolution and reduces the number of filters by one order of magnitude, with the highest number equal to 144 resulting in a much smaller number of trainable parameters and FLOPs count.

### 5.4. Model Modification and Performance (Ablation Study)

To further investigate features of Mobile-PolypNet, we tried several of its variations. Table 4 summarizes different model architectures and their performances on the Kvasir dataset. In the first variation (Mobile-Polypnet + MaxPool), in the inverted residual block, we replaced each stride-2 convolution with a stride-1 convolution followed by maxpooling. We also replaced upsampling transpose convolution with interpolated upsampling. Direct connection between the encoder and decoder in the Mobile-PolypNet backbone is the simplest form of skip connection. In the next variation we replaced the skip connection with a single convolution operation (Mobile-PolypNet + ConvSkip). This extra block increased the FLOPs count. It also took longer for the model to converge. However, an improvement in the accuracy was observed. In the next variation (Mobile-PolypNet + PT), we used the MobileNetV2 [26] pre-trained with the ImageNet dataset from the Keras library as our encoder. The decoder remained the same. We observed that although the model converged quickly, it suffered from overfitting. To reduce overfitting, we inserted dropout layers in between convolution layers (Mobile-PolypNet + Dropout) in the Mobile-PolypNet backbone. Although it converges quickly, the achieved DICE score was lower compared to other models.

As the average DICE score for five models presented in Table 4 is different, we did *t*-tests to measure the significance. Although addition of the convolution skip connection produced the highest accuracy, the difference is not significant (*p*-value = 0.815). The use of maxpooling for dimension reduction compared to stride-2 convolution and interpolation compared to transpose convolution is highly debated in the literature [29,30]. In our model, we observed a significant (*p*-value = 0.018) reduction in accuracy due to the use of maxpooling. The additional parameters required by the stride-2 and transpose convolution help to learn and preserve important spatial features in the network which improves the performance.

### 5.5. Model’s Limitations

Although our model achieved state-of-the-art accuracy, we observed that it failed to properly segment the polyp in some images. It also wrongly segmented certain blobs as polyps in some images. However, we believe by processing video frames and comparing two consecutive frames, we can reduce incorrect segmentation in some images.

## 6. Conclusions

In this paper, we presented a novel Mobile-PolypNet architecture for automatic segmentation of the colorectal polyp. The model has been tested on five publicly available datasets and compared with the current state-of-the-art models. The network achieved state-of-the-art accuracy with the orders of magnitude reduction in the computational cost. Compared with the current state-of-the-art Pranet, Mobile-PolypNet requires 83 times fewer parameters and is about 17 times more computationally efficient, making it an excellent model for a segmentation backbone for deployment on resource-sensitive devices.

## Figures and Tables

**Table 1:** Model’s performance and comparison with other models on the test dataset. Results have been reported from the PraNet [4] paper and have not been verified. The bold is to identify the best of the column.

Dataset	Kvasir				CVC-ClinicDB			
**Models**	**DICE**	**mIoU**	**F2**	**MAE**	**DICE**	**mIoU**	**F2**	**MAE**
U-Net	0.818	0.746	0.794	0.055	0.823	0.755	0.811	0.019
U-Net++	0.821	0.743	0.808	0.048	0.794	0.729	0.785	0.022
ResUNet-mod	0.791	n/a	n/a	n/a	0.779	n/a	n/a	n/a
ResUNet++	0.813	0.793	n/a	n/a	0.796	0.796	n/a	n/a
SFA	0.723	0.611	0.670	0.075	0.700	0.607	0.647	0.042
PraNet	0.898	0.840	0.885	**0.030**	0.899	0.849	0.896	0.009
**Mobile-PolypNet **	**0.935**	**0.888**	**0.894**	0.031	**0.945**	**0.906**	**0.870**	**0.008**

**Table 2:** Model’s accuracy comparison on the unseen test dataset CVC-300, Colon-DB, and ETIS. The bold is to identify the best of the column.

Dataset	CVC-300			Colon-DB			ETIS		
**Models**	**DICE**	**mIoU**	**MAE**	**DICE**	**mIoU**	**MAE**	**DICE**	**mIoU**	**MAE**
U-Net	0.710	0.627	0.022	0.512	0.044	0.061	0.398	0.335	0.036
U-Net++	0.707	0.624	0.018	0.483	0.410	0.064	0.401	0.344	0.035
SFA	0.467	0.329	0.065	0.469	0.347	0.094	0.297	0.217	0.109
PraNet	0.871	0.797	**0.010**	0.709	0.640	0.045	0.628	0.567	0.031
**Mobile-PolypNet**	**0.901**	**0.864**	0.016	**0.867**	**0.728**	**0.038**	**0.826**	**0.728**	**0.024**

**Table 3:** Model efficiency is measured in terms of the number of parameters required by the model and the number of FLOPs performed by the model to process a single image of dimension 352×352 (this image size was only used for the FLOPs count). The FLOPs count has been tested on TensorFlow, and accuracy metrics comparison were made on the Kvasir dataset. The bold is to identify the best of the column.

Models	Number of Parameters	Disk Space	FLOPs Count	DICE	mIoU	MAE
U-Net (MICCAI’15)	7.85 M	30 MB	52.6 G	0.818	0.746	0.055
U-Net++ (TMI’19)	9.04 M	34.6 MB	112.6 G	0.821	0.743	0.048
ResUNet-mod	7.85 M	30 MB	52.6 G	0.791	n/a	n/a
ResUNet++	9.04 M	34.6 MB	112.6 G	0.813	0.793	n/a
SFA (MICCAI’19)	25.59 M	97.7 MB	222.4 G	0.723	0.611	0.075
PraNet (MICCAI’20)	20.52 M	78.4 MB	81.9 G	0.898	0.840	**0.030**
**Mobile-PolypNet**	**246 K**	**1.72 MB**	**4.9 G**	**0.935**	**0.888**	0.031

**Table 4:** Computation and accuracy performance comparison of different modified models based on the same Mobile-PolypNet backbone architecture on the Kvasir dataset. FLOPs have been calculated for an image dimension of 224 × 224. The bold is to identify the best of the column.

Mobile-PolypNet Model	Number of Trainable Parameters	Number of Non-Trainable Parameters	FLOPs Count	Number of Epochs to Converge	DICE	MAE
Mobile-PolypNet	233,001	13,616	2.0 G	145	0.935	0.031
Mobile-PolypNet + MaxPool	223,913	13,616	1.8 G	217	0.900	0.047
Mobile-PolypNet + ConvSkip	250,601	13,616	2.2 G	186	**0.938**	**0.028**
Mobile-PolypNet + PT	234,618	2,495,257	**1.5 G**	**50**	0.912	0.037
Mobile-PolypNet + Dropout	233,001	13,616	2.0 G	110	0.928	0.035

## Data Availability

Code and data related to this research is available on our GitHub. https://github.com/rkarmaka/Mobile-PolypNet (accessed on 13 December 2021).
